# Work stress and burnout among young public health workers: a mechanism-based analysis of emotional labor and organizational support

**DOI:** 10.3389/fpubh.2025.1748555

**Published:** 2026-01-16

**Authors:** Shiyao Yin, Chunming Chen

**Affiliations:** 1 Henan Provincial Center for Disease Control and Prevention, Zhengzhou, China; 2 School of Culture, Tourism and Public Management (School of Special Education), Guangzhou City Polytechnic, Guangzhou, China

**Keywords:** burnout, emotional exhaustion, emotional labor, job demands, public health workforce, young workers

## Abstract

**Background:**

Burnout is an increasing concern in public health, particularly among young workers entering frontline roles with limited experience and high early workload pressure.

**Methods:**

We surveyed 410 young public health workers, including newly hired employees and volunteers in township clinics. Standardized measures assessed job demands, emotional labor, emotional exhaustion, and burnout. Structural equation modeling and bootstrap mediation analysis were conducted, and group comparisons examined differences by employment status.

**Results:**

Job demands were strongly associated with emotional exhaustion (β = 0.477, *p* < 0.001) but were not significantly related to emotional labor (β = 0.026, *p* = 0.607). Emotional labor was positively associated with emotional exhaustion (β = 0.512, *p* < 0.001). Emotional exhaustion showed the strongest association with burnout (β = 0.675, *p* < 0.001), while the direct path from emotional labor to burnout was not significant (β = 0.075, *p* = 0.075). Bootstrap tests supported a significant indirect effect of job demands on burnout through emotional exhaustion (effect = 0.307, 95% CI [0.245, 0.375], *p* < 0.001), whereas indirect effects involving emotional labor were not supported. Newly hired employees reported higher levels of emotional exhaustion and burnout than volunteers.

**Conclusions:**

Early burnout risk among young public health workers appears to be driven primarily by sustained exhaustion linked to job demands. Preventive efforts should prioritize realistic workload arrangements, clear role expectations, and stable organizational conditions that limit exhaustion accumulation during early career stages.

## Introduction

### Global concern over the escalation of stress and burnout in public health work environments

Public health systems in many countries have been operating under pressure for years, and the weight of this accumulated strain is increasingly visible in day-to-day work. Burnout, as described by Maslach et al. (1), involves emotional depletion, a sense of distancing from work, and a decline in perceived professional capability. Their work emphasized that these reactions do not arise suddenly but build up when individuals spend long periods coping with persistent stress. Demerouti et al. (2) reached a similar conclusion from a different angle by showing how sustained mismatches between job demands and available resources gradually weaken workers' ability to adjust, allowing burnout to take hold over time. Findings from several countries point to the same direction. Rotenstein et al. (3), working with physician samples from varied health systems, reported that high burnout levels appeared regardless of national context or institutional arrangements. Research with nursing staff adds another layer. Woo et al. (4) found that emotional labor, rotating work schedules, and chronic staffing shortages together created conditions in which exhaustion accumulated quickly. During the COVID-19 period, the situation intensified. Ghahramani et al. (5) noted that heavier duties, infection risk, and heightened emotional stress placed frontline workers under pressures that exceeded what was common even in already demanding environments. More recent assessments of the public health workforce suggest that this pattern has not eased. Nagarajan et al. (6) describe burnout as a persistent issue, particularly in roles that require direct community engagement or rapid response to crises. The consequences extend beyond reduced energy or motivation. Salvagioni et al. (7), drawing on longitudinal evidence, showed that prolonged burnout is tied to deteriorations in both physical and mental health, higher intentions to leave the job, and disruptions in work performance. When placed together, these findings point to a sector where long-standing structural strain is shaping the everyday experiences of public health workers and where burnout reflects not only individual fatigue but also deeper pressures within the systems in which they work.

### Developments in burnout theory and approaches to occupational health intervention

The study of burnout has been shaped by several influential lines of work rather than a single linear progression. One early contribution came from Hobfoll (8), who treated stress not simply as a reaction to external demands but as a process in which people lose resources faster than they can replace them. His argument highlighted why exhaustion accumulates in situations where individuals face repeated setbacks or lack opportunities to regain what they expend. Later efforts shifted toward refining how burnout is defined and measured. Kristensen et al. (9), in developing the Copenhagen Burnout Inventory, drew attention to variation in the sources of exhaustion and offered a structure that allowed researchers to examine these origins separately. Shirom and Melamed (10) added further detail by comparing major assessment tools and describing burnout through emotional, physical, and cognitive expressions, which helped clarify the boundaries of the construct for empirical work. Research gradually moved closer to the conditions under which people work. Schaufeli et al. (11) showed that exhaustion increases when work becomes heavier while the means to cope diminish, placing burnout within the fabric of organizational life rather than solely in the individual. Questions about how to intervene became more prominent around the same time. Nielsen and Randall (12) argued that evaluating workplace interventions requires attention to how they are carried out and to the settings in which they unfold, noting that outcomes alone do not explain whether an intervention is functioning as intended. Montano et al. (13) reached a similar conclusion in their review of organizational programs, pointing out that many initiatives fall short when their design does not match the realities of the work environment or when implementation is inconsistent. The boundaries between burnout and related forms of distress also received scrutiny. Bianchi et al. (14) examined the extent to which burnout overlaps with depression and raised questions about the distinctiveness of the concept, prompting further discussion of the mechanisms through which chronic work strain affects mental health. These contributions, taken from different strands of research, now form much of the conceptual backdrop for studies of workplace strain. They also offer practical direction for building intervention strategies that reflect the demands, constraints, and pressures found in contemporary public health work settings.

### Limitations in current burnout intervention research and the need for a more coherent framework for public health settings

Research on burnout interventions has grown rapidly, yet findings from recent reviews suggest that many of the approaches now in use do not fully match the realities of public health work. Bes, Shoman et al. (15), reviewing programs implemented in organizational settings, found that reductions in emotional exhaustion were often small. Their analysis showed that these programs tended to rely on narrow strategies that operated in isolation from the structural conditions shaping everyday work. Cohen et al. (16), surveying interventions used with nurses, physicians and other health professionals, reached a similar conclusion. They observed that many initiatives focused primarily on personal skills or emotional coping, while issues linked to staffing, workflow or resource availability received comparatively little attention. As a result, several programs showed initial benefits that proved difficult to maintain once they were introduced into busy clinical environments. Work carried out with high-pressure occupations points in the same direction. In a realist review, Taylor et al. (17) showed that the pressures driving burnout among nurses, midwives and paramedics differed markedly across organizations and work contexts. Their findings illustrated why interventions built around general principles rarely function as intended when the conditions that create strain vary so widely. Important questions also remain about how burnout develops within different organizational settings. Drawing on longitudinal evidence, Shoman et al. (18) argued that emotional exhaustion often reflects multiple forces acting together rather than a single strain pathway. Yet many existing models capture only a part of this interaction, leaving uncertainty about how these processes unfold in different environments. The realist synthesis tradition offers a way to address this gap. Rycroft-Malone et al. (19) emphasized the need to identify the specific processes through which an intervention is expected to operate and the circumstances that shape these processes. Wong et al. (20) added further detail to this approach by clarifying how researchers can trace the links between context, mechanism and outcome in complex workplace interventions. Evaluation research has also moved toward a more structured examination of how interventions take shape in practice. Moore et al. (21) highlighted the importance of attending to how programs are introduced, how they interact with existing routines and how their effects may differ across levels of an organization. These developments, considered together, indicate that burnout within public health settings is influenced by resource conditions, work organization and the psychological demands placed on workers, yet few intervention models address these elements within a single framework. Building on this body of work, the present study proposes a model designed for the specific pressures found in public health systems, linking stress exposure, organizational support and individual psychological responses in a structure that can guide the development of interventions suited to complex and highly variable work environments.

## Review

### Stress structures and burnout risk in public health work environments

Work in public health often unfolds under a set of pressures that accumulate steadily over time. Many positions involve a wide mix of administrative and field tasks, heavy coordination duties and emotionally charged interactions with the public. These pressures became easier to observe during the early stage of the COVID-19 response. Stone et al. (22), describing the daily routines of staff involved in surveillance and communication work, noted that workers were asked to update case information, relay policy instructions and handle public enquiries under conditions that left very little room to pause or reorganize their workload. Scales et al. (23), examining a later stage of the response, found that the same kinds of demands persisted for long stretches, and many staff reported fatigue and a gradual loss of psychological reserves rather than the stabilization one might expect as systems adjusted to the crisis. Studies focused on emotional demands draw a similar picture. Kim et al. (24) described how public health nurses spent much of their time dealing with anxious residents and clarifying policy shifts, often in situations where they had to manage tension or conflict without the benefit of formal support structures. Work of this kind frequently affects sleep patterns and overall energy. Baek et al. (25) found that staff who engaged in frequent emotionally charged conversations had more difficulty recovering at the end of the workday, and many experienced disruptions in rest that carried into subsequent shifts. Resource limitations add another layer to this environment. Forte et al. (26), studying community health roles, pointed out that many workers carried service responsibilities that exceeded available staffing or equipment, and the resulting imbalance made it harder to maintain a stable sense of wellbeing. A parallel situation was documented in Poland. Izdebski et al. (27) described extended work hours, shortages of essential materials and persistent gaps in organizational backing, all of which contributed to reports of feeling overloaded early in the work cycle. Longer-term studies of burnout mechanisms help explain why these pressures accumulate in the way they do. Shoman et al. (18), using multi-wave data, noted that emotional exhaustion tends to build when workers have few opportunities to step back or recalibrate their effort, and when periods of strain follow one another without interruption. The conditions they identify—ongoing resource depletion and limited recovery time—closely resemble the work patterns observed in many public health roles. Research conducted across different regions and job categories suggests that the combination of heavy task demands, steady emotional strain and recurring resource shortages has become a routine feature of public health work. Under such circumstances, burnout risk is less an individual aberration than a foreseeable consequence of extended exposure to these working conditions.

### Theoretical foundations and mechanisms underlying occupational burnout

Work on burnout first developed around attempts to clarify what workers actually experience when exhaustion takes hold. Maslach and Jackson (28) described emotional exhaustion as the clearest indication of this state and treated burnout as a gradual loss of emotional resources that builds during periods of unrelieved strain. As the field broadened, researchers studying emotional labor began to explain how specific work conditions shape this depletion. Morris and Feldman (29) showed that many workers must present particular emotional expressions during interactions with clients, often in situations where the volume of such encounters gives little time to reset between them. Grandey (30) distinguished between different ways of managing these demands and found that efforts aimed only at outward display require workers to suppress their own reactions repeatedly, a pattern linked with sharper declines in emotional energy. Brotheridge and Grandey (31) compared these regulation styles across varied job settings and observed that the strain produced by each method depends heavily on the work environment. Zapf (32) added that expectations surrounding displays of emotion are embedded in interaction routines and role structures, which makes emotional labor a steady source of pressure rather than an occasional requirement. Another stream of research examined how burnout relates to broader processes of energy loss. Demerouti et al. (33) used multiple assessment tools to show that indicators of exhaustion tend to cluster with other signs of diminished energy, supporting the view that burnout reflects a sustained drain rather than a short-term reaction. Shirom (34) approached the issue by describing burnout as a decline across emotional, physical and cognitive functioning, and noted that such declines are more pronounced in roles where demands continue with little variation. Halbesleben's (35) meta-analysis, drawing on conservation of resources theory, indicated that workers often lose resources in a chain-like fashion: once demands rise and emotional labor becomes heavier, chances for recovery narrow, and the next period of work begins before energy has returned. Seen across these lines of inquiry, burnout has been examined from several angles: as an emotional condition, as a form of strain rooted in daily interpersonal demands, and as a pattern of resource loss that accelerates under continuous pressure. Despite differences in emphasis, these studies point toward exhaustion as the most stable indicator of burnout and show how repeated demands and limited recovery gradually reduce workers' capacity to stay engaged. This body of work helps explain why burnout develops in roles characterized by sustained pressure and provides a basis for investigating how organizational conditions shape the course of this erosion.

### Collaborative evidence on organizational support, job characteristics, and individual psychological mechanisms

Research on burnout increasingly points to the ways workplace conditions shape how strain develops. Early work on perceived organizational support offered one of the first explanations for this connection. Eisenberger et al. (36) showed that when employees believe their organization values their contribution, demanding workloads are less likely to translate into emotional strain. Rhoades and Eisenberger's (37) review added evidence from a wide range of occupations and noted that support from the organization influences how workers interpret pressure and whether they remain engaged during demanding periods. In a later synthesis, Kurtessis et al. (38) found that support systems that operate reliably over time help workers retain a sense of stability even in jobs where workload fluctuations are common. Studies of interpersonal dynamics inside organizations describe related mechanisms. Edmondson (39) argued that employees' willingness to speak openly about problems depends on whether they feel safe doing so, a feature especially relevant in public health work where new situations can arise without warning. Leadership practices influence this environment in clear and subtle ways. Gerstner and Day (40) reported that workers with stronger relationships with their supervisors were more likely to receive emotional reassurance and practical guidance when facing demanding tasks. Skakon et al. (41) later showed that leaders' emotional states often shape employees' reactions to stressful situations. Thomas et al. (42) observed that workers frequently draw cues from leaders when trying to make sense of ambiguous or competing job expectations. Work examining job structure provides another angle on burnout risk. Xanthopoulou et al. (43) found that workers' psychological strengths affect how they interpret the pressures that accompany demanding tasks and can reduce the likelihood that strain will intensify over time. Building on this tradition, Tims et al. (44) described how employees sometimes adjust aspects of their work to manage pressure more effectively. Their findings suggest that people are capable of altering parts of their job to protect their energy when formal support or other resources are limited. Across these areas of inquiry, burnout appears to develop from the combined influence of organizational conditions, team interactions, job structure and individual capacities. In public health settings, where responsibilities shift quickly, emotionally charged encounters are common and organizational systems are often layered, these influences frequently occur at the same time. This combination helps explain why workers in such settings face enduring pressure and why a prevention model must consider several points at which strain accumulates or eases.

### International practices in occupational health interventions and emerging directions for integrated prevention models

Research on occupational health interventions often points to the role that workplace structures play in shaping whether programs are effective. Anger et al. (45), reviewing mental health programs for healthcare workers, found that many initiatives address skills, support or resources but vary widely in how well they fit with existing organizational arrangements. Their findings suggested that the strength of the underlying organizational system determines whether programs remain useful once initial enthusiasm fades. Cooklin et al. (46), working from an occupational health and safety perspective, reached a similar conclusion. They argued that policies, procedures and everyday job conditions need to work together, because efforts introduced in isolation tend to lose momentum. This idea appears as well in research on Total Worker Health. Feltner et al. (47) noted that programs grounded in clear administrative structures and stable resource pathways were more likely to show sustained effects. Studies conducted at the organizational level in healthcare illustrate similar patterns. Gray et al. (48) described how workers faced a combination of emotional strain, heavy task requirements and procedural limits that restricted recovery. Under such conditions, interventions focused solely on coping skills produced small and short-lived gains. Other forms of intervention offer a different view. Roczniewska et al. (49), drawing on randomized controlled trials, reported improvements when employees were encouraged to reshape aspects of their work or redistribute their effort to manage day-to-day pressure. Sakuraya et al. (50) observed comparable outcomes in settings where employees were given room to adjust their tasks and seek access to needed resources. These findings speak to the conditions often seen in public health services, where young workers and recent graduates commonly move between assignments with little certainty and rely on small adjustments in daily work to maintain some sense of balance. Attention has also turned to the difficulty many organizations face in diagnosing the problems they hope to address. Nebbs et al. (51) found that institutions frequently lack tools to assess needs or constraints, which leads to interventions that do not match existing conditions. Integrated approaches provide a clearer way to understand this challenge. Dennerlein et al. (52), working with the Total Worker Health framework, explained how safety practices, communication routines, task requirements and opportunities for recovery often reflect one another within high-risk workplaces. Nielsen and Christensen (53) added that workers' experiences, team interactions, management practices and broader organizational systems can shift together in ways that influence health-related outcomes. Evaluations of participatory interventions also highlight the practical limits of implementation. Arapovic-Johansson et al. (54) described repeated difficulties in establishing stable participation, noting that context and timing often determine whether an initiative takes hold. Across these areas of evidence, researchers have begun to move away from stand-alone training or narrowly targeted programs. Public health work presents a particular challenge because responsibilities can shift abruptly, emotional encounters are common and the support available to younger workers is uneven. Under these conditions, existing interventions often fall short of providing reliable protection against strain. These limitations form the basis for the model developed in this study, which brings organizational conditions, job pressures, psychological resources and available support into one structure suited to the realities of public health work and used to guide the study's hypotheses.

## Methodology

### Study design and participants

This study used a cross-sectional survey to examine how work-related strain develops among young public health workers and to clarify the roles of job demands, emotional labor, emotional exhaustion, and burnout in the early stage of public health work. Job demands were conceptualized based on the Job Demands–Resources framework, which identifies job demands as work characteristics that require sustained effort and are linked to strain outcomes such as exhaustion and burnout (2, 55). Emotional labor was measured with validated self-report instruments adapted from existing emotional labor scales such as the Chinese context emotional labor scale developed by Yang et al. (56), which captures surface acting, deep acting and other dimensions of required emotion regulation at work. Burnout and emotional exhaustion were assessed using established measures drawn from the Maslach Burnout Inventory tradition, focusing on emotional exhaustion as the core component of burnout (57).

The study targeted individuals who had recently entered public health practice and included two groups: university students working as volunteers in township or community health settings and newly hired employees employed in public health agencies. Volunteers were mainly involved in resident communication, basic data collection, and health promotion activities that required frequent interpersonal interaction but were often carried out with limited formal organizational support. Newly hired employees worked within more structured administrative systems and clearer role expectations, yet many were still adjusting to growing workloads and emotionally demanding responsibilities. Data were collected in cooperation with township health centers, community health service organizations, disease control units, and university practice sites, using both online and paper-based questionnaires. All participants were informed about the purpose of the study, confidentiality arrangements, and the voluntary nature of participation, and informed consent was obtained. A total of 410 valid questionnaires were included in the analyses. The sample was evenly divided between volunteers (50.7%) and newly hired employees (49.3%), with a moderately balanced gender distribution (58% and 42%). Participants ranged in age from 19 to 30 years (M = 24.4, SD = 3.51), and the age distribution was relatively even across three groups–18–22 years, 23–26 years, and 27–30 years—capturing several early-career stages. This sample size was sufficient for the planned structural equation modeling, mediation analyses, and subgroup comparisons. Employment role, gender, and work context were included as background variables to help distinguish differences in overall strain levels from differences in the underlying stress processes experienced by volunteers and newly hired employees.

### Indicator framework

**Figure 1:**
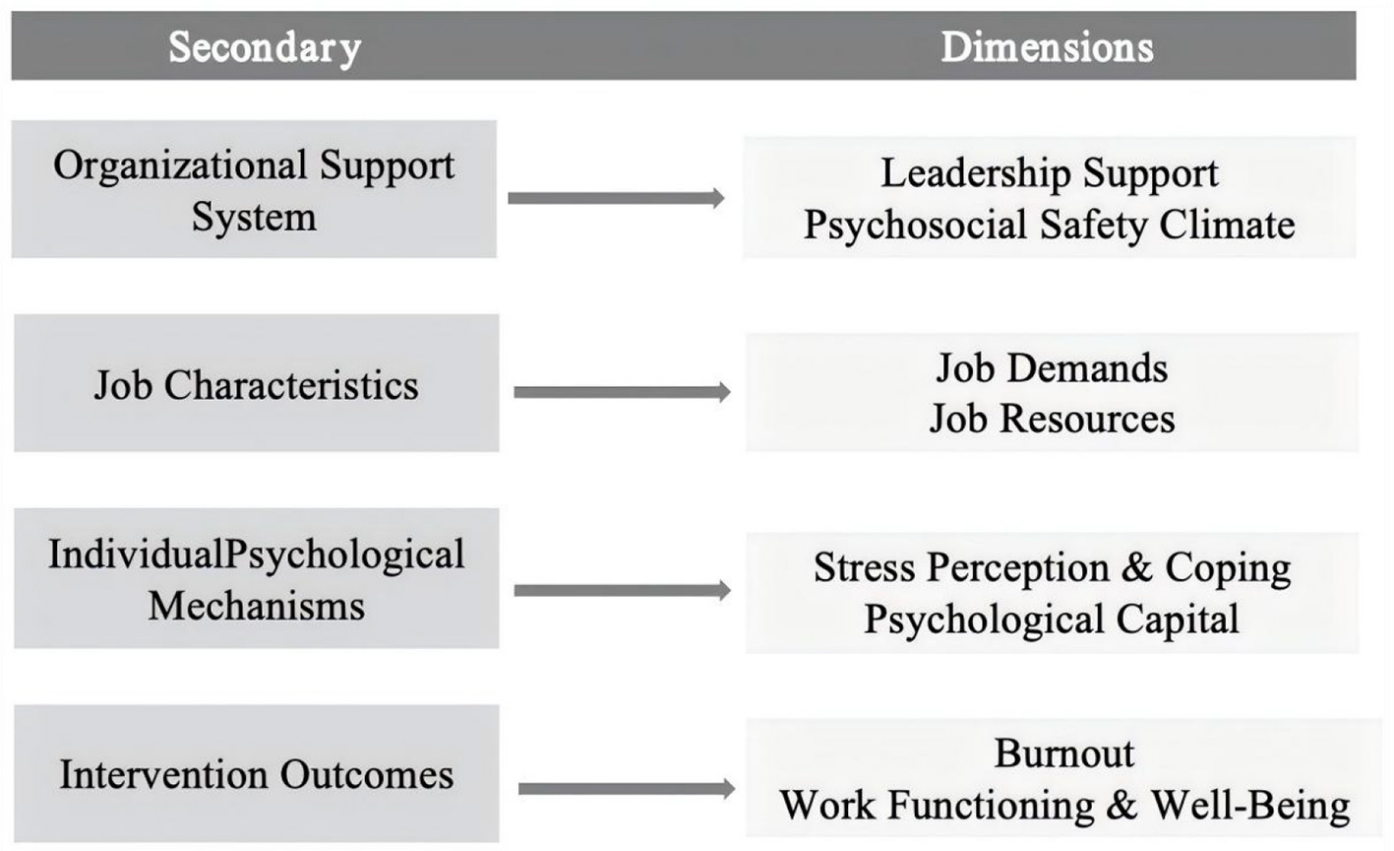
Indicator system for organizational support, job characteristics, individual 293 psychological mechanisms, and intervention outcomes.

Figure 1 shows the indicator framework that guided the questionnaire design and the organization of variables in this study. Based on this framework, the questionnaire covered factors at the organizational, job, and individual levels that are relevant to burnout among young public health workers. Organizational support was measured through leadership support and psychosocial safety climate, reflecting how much guidance, protection, and support workers receive from their institutions. Job characteristics were captured through job demands and job resources, focusing on workload pressure and available work-related support. At the individual level, psychological mechanisms included stress perception, coping, and psychological capital, which describe how individuals experience and manage emotionally demanding situations. Burnout and work functioning and wellbeing were treated as the main outcome indicators. All measures were drawn from commonly used scales in occupational health and organizational research, with minor adjustments to fit the public health context. This framework helps clarify how the questionnaire variables are linked and provides the basis for the analyses reported later in the paper.

## Results

### Measurement model: reliability and validity

Before testing the structural relationships, we first examined whether the measurement instruments functioned adequately in this sample. As reported in Table 1, all constructs showed satisfactory internal consistency, with Cronbach's α values ranging from 0.868 to 0.920, indicating stable reliability across scales. Composite reliability estimates were similarly high, and all average variance extracted (AVE) values exceeded commonly accepted thresholds, suggesting that the items within each construct shared sufficient variance and that convergent validity was acceptable. The descriptive statistics show moderate mean levels for all variables, with reasonable dispersion, indicating no evidence of extreme skewness or restriction of range. In addition, the confirmatory factor analysis supported the adequacy of the measurement structure. The overall model fit indices indicated a good fit between the measurement model and the observed data (χ^2^ = 252.98, df = 237; CFI = 0.998; TLI = 0.997; RMSEA = 0.013; SRMR = 0.026). Although volunteers and newly hired employees differed in their roles and in reported levels of strain, the measurement model performed consistently at the sample level. Overall, these results suggest that the measures used in this study demonstrated sound reliability and validity, providing a suitable basis for subsequent analyses of the structural relationships among job demands, emotional labor, emotional exhaustion, and burnout.

**Table 1:** Descriptive statistics and measurement properties.

**Variable**	**Mean**	**SD**	**Cronbach's α**
Job Demands (JD)	3.15	0.72	0.898
Emotional Labor (EL)	3.16	0.63	0.868
Organizational Support (OS)	3.29	0.79	0.920
Psychological Capital (PC)	2.96	0.74	0.915
Emotional Exhaustion (EXH)	3.14	0.72	0.901
Burnout (BUR)	2.93	0.69	0.891

### Structure of work demands and stressors

As shown in Table 2, differences between volunteers and newly hired employees are modest in magnitude but display a consistent pattern across key variables. Volunteers report lower mean levels of job demands (M = 3.00) compared with newly hired employees (M = 3.30). A similar tendency is observed for emotional labor, with volunteers showing slightly lower average scores (M = 3.12) than newly hired employees (M = 3.21), although this difference does not reach statistical significance. Clearer group differences emerge for emotional exhaustion and burnout. Newly hired employees report higher levels of emotional exhaustion (M = 3.33) and burnout (M = 3.15), whereas volunteers report lower corresponding levels (M = 2.97 and M = 2.71, respectively). Overall, these results suggest that newly hired employees experience higher levels of work-related strain during the early stage of their public health work, while volunteers report comparatively lower strain across most indicators.

**Table 2:** Group differences in work demands and stress indicators.

**Variable**	**Volunteers M(SD)**	**New employees M(SD)**	***t***	***p***
Job Demands (JD)	3.00 (0.68)	3.30 (0.74)	−4.23	< 0.001
Emotional Labor (EL)	3.12 (0.69)	3.21 (0.57)	−1.57	0.118
Emotional Exhaustion (EXH)	2.97 (0.66)	3.33 (0.72)	−5.34	< 0.001
Burnout (BUR)	2.71 (0.62)	3.15 (0.68)	−6.82	< 0.001

### Structural relations between job demands and burnout dimensions: an SEM analysis

**Figure 2:**
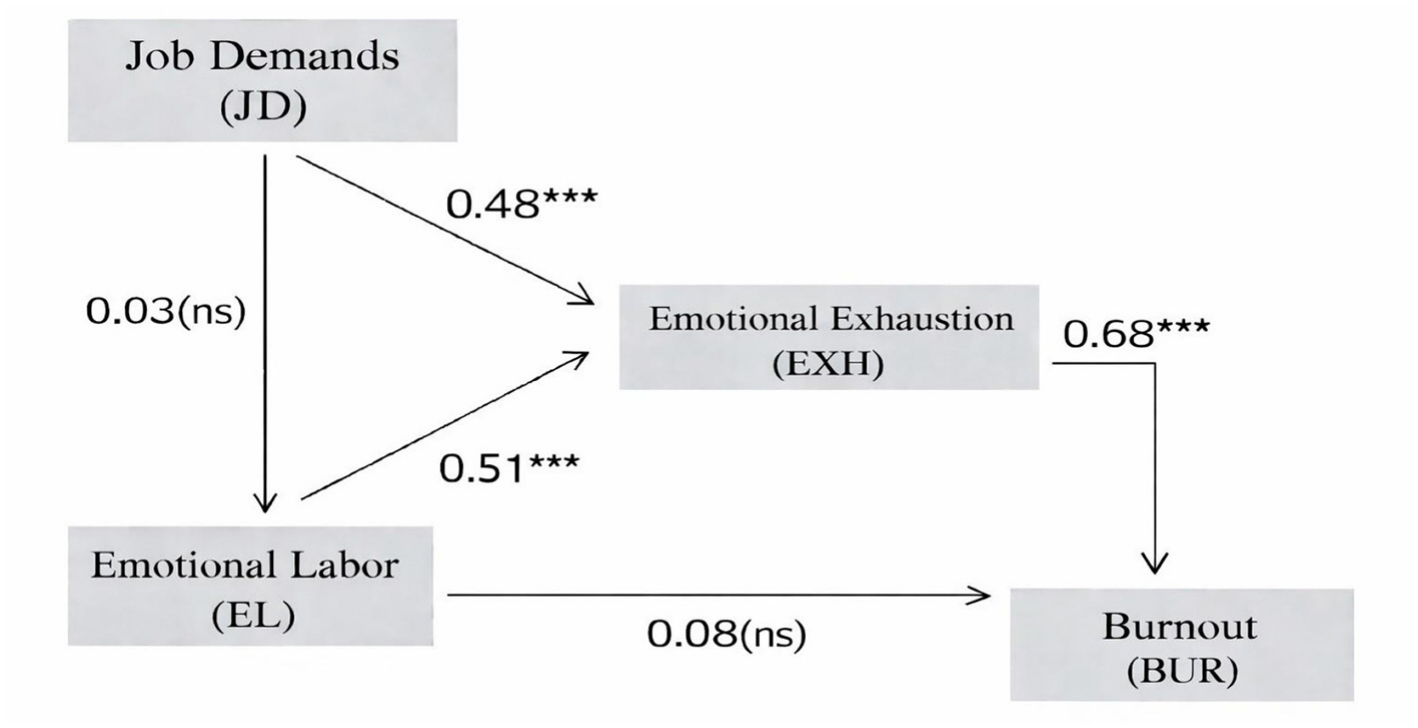
Structural equation model of job demands, emotional labor, emotional exhaustion, and burnout. ^***^Indicates that the results are statistically significant at the *p* < 0.001 level.

Across Tables 3, 4 and Figure 2, the results depict a set of interconnected paths linking job demands, emotional labor, emotional exhaustion, and burnout. Job demands show a strong positive association with emotional exhaustion (β = 0.477, *p* < 0.001), whereas their association with emotional labor is not significant (β = 0.026, *p* = 0.607). Emotional labor is positively related to emotional exhaustion (β = 0.512, *p* < 0.001). Emotional exhaustion, in turn, shows a strong association with burnout (β = 0.675, *p* < 0.001). The direct path from emotional labor to burnout is not statistically significant (β = 0.075, *p* = 0.075). Together, the model explains 50.3% of the variance in emotional exhaustion and 51.4% of the variance in burnout. As illustrated in Figure 2, emotional exhaustion occupies a central position in the model, linking work demands and emotional labor to burnout. Overall, the results indicate a stable pattern of associations among these variables in the sample of young public health workers.

**Table 3:** Structural equation model: path coefficients and variance explained (R^2^).

**Path**	**β**	**SE**	***p***	**Sig**
JD → EL	0.026	0.044	0.607	
JD → EXH	0.477	0.038	< 0.001	^***^
EL → EXH	0.512	0.041	< 0.001	^***^
EXH → BUR	0.675	0.040	< 0.001	^***^
EL → BUR	0.075	0.046	0.075	

^***^Indicates that the results are statistically significant at the *p* < 0.001 level.

**Table 4:** Variance explained (R^2^).

**Construct**	**R^2^**
Emotional Exhaustion (EXH)	0.503
Burnout (BUR)	0.514

### Mediation of emotional exhaustion

Table 5 presents the bootstrap results for the indirect effects linking job demands to burnout among young workers. The indirect path from job demands to burnout through emotional exhaustion is statistically significant (effect = 0.307, 95% CI [0.245, 0.375], *p* < 0.001), indicating that emotional exhaustion serves as a key mechanism through which job demands are associated with burnout. In contrast, the indirect paths involving emotional labor are not significant. Specifically, the indirect effect of job demands on emotional exhaustion through emotional labor is small and non-significant (effect = 0.013, *p* = 0.613), and the indirect effect of job demands on burnout through emotional labor alone is also non-significant (effect = 0.002, *p* = 0.678), with confidence intervals including zero. Taken together, these results indicate that the association between job demands and burnout operates primarily through emotional exhaustion, rather than through multiple parallel mediating pathways involving emotional labor.

**Table 5:** Bootstrap indirect effects.

**Indirect Path**	**Effect**	**SE**	**95% CI LL**	**95% CI UL**	***p***	**Sig**
JD → EXH → BUR	0.307	0.033	0.245	0.375	< 0.001	^***^
JD → EL → EXH	0.013	0.026	−0.037	0.066	0.613	
JD → EL → BUR	0.002	0.004	−0.005	0.013	0.678	

^***^Indicates that the results are statistically significant at the *p* < 0.001 level.

### Overall fit of the integrated burnout prevention model

Table 6 provides evidence that the proposed model offers a reasonable representation of the relationships among work demands, emotional labor, emotional exhaustion, and burnout in the sample of young public health workers. Because the model is based on a small set of observed variables and includes closely related pathways, overall model adequacy was assessed with attention to both model parsimony and residual fit. Comparisons across alternative specifications indicate that the final, more parsimonious structure achieves a better balance between explanatory power and simplicity. Supplementary fit indicators further suggest that the model captures the observed patterns with acceptable accuracy. Taken together, the results support the view that the associations among work demands, emotional effort, exhaustion, and burnout follow a coherent and stable structure rather than reflecting scattered or isolated stress responses. The model therefore provides a sound basis for understanding how early work conditions shape the accumulation of strain among young public health workers, highlighting the role of job organization and emotionally demanding tasks in the development of burnout.

**Table 6:** Overall model fit indices.

**Model**	**AIC**	**BIC**	**ΔAIC**	**ΔBIC**
Final Parsimonious Model	1,971.94	2,012.10	0.00	0.00
Full Structural Model	1,973.66	2,017.84	1.73	5.74
Reduced Direct-Effect Model	1,975.09	2,015.25	3.16	3.15

### Subgroup analysis of young volunteers and early-career employees

Table 7 examines whether volunteers and newly hired employees differ in the way work demands, emotional labor, emotional exhaustion, and burnout are connected. Although the two groups differ in their overall levels of strain, additional analyses indicate that the structural relationships among these variables are largely comparable across employment roles. Multi-group tests show that constraining the key paths to be equal between volunteers and newly hired employees does not significantly reduce model fit, suggesting that the basic mechanism through which work demands are linked to emotional exhaustion and burnout operates similarly in both groups. At the same time, employment status is associated with differences in the intensity of experienced strain. Newly hired employees report higher levels of emotional exhaustion and burnout than volunteers, even after accounting for the core structural paths, indicating that group differences emerge primarily at the level of sustained strain rather than in the form of distinct psychological processes. Taken together, these findings suggest that while volunteers and new employees follow a similar pathway through which work pressure accumulates, newly hired employees are positioned at a higher point along this pathway, making them more vulnerable to burnout. This pattern highlights the importance of focusing prevention efforts on reducing prolonged exhaustion and improving working conditions during the early stages of formal employment in public health settings.

**Table 7:** Effects of employment status on emotional labor, emotional exhaustion, and burnout.

**Path**	**β**	**SE**	***p***
Role → EL	0.075	0.063	0.134
Role → EXH	0.124	0.051	< 0.001
Role → BUR	0.153	0.047	< 0.001

## Discussion

### Early role load and burnout risk in the context of existing burnout research

The present findings are largely consistent with established burnout theories and empirical evidence in occupational and health psychology. Classic work by maslach et al., (57) conceptualized burnout as a response to chronic job stress, with emotional exhaustion as its core component. In line with this framework, emotional exhaustion in the current study showed the strongest association with burnout, supporting its central role among young public health workers. Similar patterns have been repeatedly observed across healthcare professions, including physicians and nurses, as documented in large-scale reviews and meta-analyses (3–5). Our results extend these findings to the public health workforce, a group that has received comparatively less empirical attention despite its growing responsibilities in recent years (6).The observed links among job demands, emotional exhaustion, and burnout are also consistent with the Job Demands–Resources (JD–R) model proposed by Demerouti et al. (2). According to this model, high job demands deplete energy and lead to exhaustion, which subsequently increases burnout risk. In the present study, In the present study, job demands were directly associated with emotional exhaustion and indirectly associated with burnout primarily through emotional exhaustion, consistent with longitudinal evidence showing that sustained demands predict exhaustion over time (11, 18), echoing longitudinal evidence showing that sustained demands predict exhaustion over time (11, 18). The relatively high proportion of explained variance in exhaustion and burnout suggests that this demand-driven process operates in a stable and systematic way among young workers at the beginning of their careers.

### Emotional labor and resource conditions in the demand–exhaustion–burnout process

Beyond confirming the Job Demands–Resources framework, the present findings clarify how emotional labor relates to the accumulation of fatigue among young public health workers, while also indicating that job demands do not necessarily translate into emotional labor in a uniform way. In the structural model, the path from job demands to emotional labor was not supported, whereas emotional labor showed a robust positive association with emotional exhaustion. This pattern suggests that interaction-related emotion regulation is closely tied to exhaustion, even when it is not directly driven by overall workload pressure as captured by the job demands measure. At the same time, job demands displayed a strong direct association with emotional exhaustion, and emotional exhaustion remained the most proximal and influential predictor of burnout. Bootstrap results further reinforced this structure: the indirect association between job demands and burnout was primarily transmitted through emotional exhaustion, while indirect paths involving emotional labor were not supported. Taken together, these results indicate that emotional labor functions as an exhaustion-relevant process in this sample, but it does not constitute the main mediating route through which job demands affect burnout. Rather, the demand–burnout link appears to operate largely through sustained exhaustion, consistent with perspectives that view exhaustion as the central channel through which chronic work pressure becomes burnout. Because moderation effects were not supported in the final models, organizational and personal resources are best interpreted as contextual factors discussed in the broader literature, but they are not empirically confirmed as buffers of the focal structural links in the present data.

### Positioning the findings within public health workforce research

Recent studies have highlighted high burnout prevalence within the public health workforce, particularly during crisis periods such as the COVID-19 response (22, 23). The present study complements this work by focusing on early-career workers and by unpacking the psychological processes that link demands to burnout. Rather than attributing burnout to individual vulnerability, the findings align with organizational-level perspectives that emphasize structural conditions and resource availability (12, 17). This emphasis is consistent with evidence from intervention research showing that organizational changes are more effective than individual-focused strategies alone in reducing exhaustion (13, 45).Overall, the present findings are largely consistent with previous research on burnout while adding specificity to the early career stage of public health work. By integrating emotional labor into the demand–exhaustion–burnout pathway and highlighting the role of organizational and personal resources as conditions associated with lower levels of strain, this study contributes to a more nuanced understanding of how burnout develops among young public health workers.

## Conclusion

This study examined burnout risk among young public health workers and tested a mechanism-based pathway linking job demands, emotional labor, emotional exhaustion, and burnout at the early career stage. The results consistently identified emotional exhaustion as the central explanatory component: job demands were strongly associated with emotional exhaustion, and emotional exhaustion showed the strongest association with burnout. Bootstrap analysis further supported that the influence of job demands on burnout operated primarily through emotional exhaustion, indicating that sustained fatigue represents the main route through which early workload pressure translates into burnout risk. Emotional labor was also closely related to emotional exhaustion, suggesting that ongoing emotion regulation during interpersonal work is an important correlate of fatigue; however, job demands did not significantly predict emotional labor, and emotional labor did not show a significant direct association with burnout. Together, these findings imply that early burnout in public health work is driven less by a broad “demands → emotional labor” chain and more by the direct accumulation of exhaustion under persistent demands, with emotional labor functioning as an exhaustion-relevant process rather than the primary mediating bridge from demands to burnout. Group comparisons further indicated that newly hired employees reported higher emotional exhaustion and burnout than volunteers, suggesting that formal entry into organizational roles may place young workers at a higher level of sustained strain even when the underlying structural relationships are similar. From a practical standpoint, prevention efforts should prioritize reducing prolonged exhaustion by addressing workload organization and recovery opportunities, clarifying role expectations during onboarding, and stabilizing daily work conditions that limit continuous depletion. Given the cross-sectional design, causal inference should be made cautiously, and future longitudinal and intervention studies are needed to determine whether modifying early workload structures and recovery conditions can effectively curb exhaustion accumulation and, in turn, reduce burnout among young public health workers.

## Data Availability

The raw data supporting the conclusions of this article will be made available by the authors, without undue reservation.

## References

[1] Maslach C Schaufeli WB Leiter MP. Job burnout. Annu Rev Psychol. (2001) 52:397–422. doi: 10.1146/annurev.psych.52.1.397 11148311

[2] Demerouti E Nachreiner F Bakker AB Schaufeli WB. The job demands–resources model of burnout. J Appl Psychol. (2001) 86:499–512. doi: 10.1037//0021-9010.86.3.499 11419809

[3] Rotenstein LS Torre M Ramos MA . Prevalence of burnout among physicians: a systematic review. JAMA. (2018) 320:1131–50. doi: 10.1001/jama.2018.12777 30326495 PMC6233645

[4] Woo T Ho R Tang A Tam W. Global prevalence of burnout symptoms among nurses: a systematic review and meta-analysis. J Psychiatr Res. (2020) 123:9–20. doi: 10.1016/j.jpsychires.2019.12.015 32007680

[5] Ghahramani S Lankarani KB Yousefi M Heydari K Shahabi S Azmand S. A systematic review and meta-analysis of burnout among healthcare workers during COVID-19. Front Psychiatry. (2021) 12:758849. doi: 10.3389/fpsyt.2021.758849 34858231 PMC8631719

[6] Nagarajan R Ramachandran P Dilipkumar R Kaur P . Global estimate of burnout among the public health workforce: a systematic review and meta-analysis. Human Resour Health. (2024) 22:44. doi: 10.1186/s12960-024-00917-w 38773482 PMC11110232

[7] Salvagioni DAJ Melanda FN Mesas AE González AD Klein CH Bocchi S. Physical, psychological and occupational consequences of job burnout: a systematic review of prospective studies. PLoS ONE. (2017) 12:e0185781. doi: 10.1371/journal.pone.0185781 28977041 PMC5627926

[8] Hobfoll SE. Conservation of resources: a new attempt at conceptualizing stress. Am Psychol. (1989) 44:513–24. doi: 10.1037/0003-066X.44.3.513 2648906

[9] Kristensen TS Borritz M Villadsen E Christensen KB. The Copenhagen Burnout Inventory: a new tool for the assessment of burnout. Work Stress. (2005) 19:192–207. doi: 10.1080/02678370500297720

[10] Shirom A Melamed S. A comparison of the construct validity of two burnout measures in two groups of professionals. Int J Stress Manag. (2006) 13:176–200. doi: 10.1037/1072-5245.13.2.176

[11] Schaufeli WB Bakker AB Van Rhenen W. How changes in job demands and resources predict burnout, work engagement, and sickness absenteeism. J Organ Behav. (2009) 30:893–917. doi: 10.1002/job.595

[12] Nielsen K Randall R. Opening the black box: presenting a model for evaluating organizational-level interventions. Eur J Work Organizat Psychol. (2013) 22:601–17. doi: 10.1080/1359432X.2012.690556

[13] Montano D Hoven H Siegrist J. Effect of organisational-level interventions at work on employees' health: a systematic review. BMC Public Health. (2014) 14:135. doi: 10.1186/1471-2458-14-135 24507447 PMC3929163

[14] Bianchi R Schonfeld IS Laurent E. Burnout–depression overlap: a review. Clin Psychol Rev. (2015) 36:28–41. doi: 10.1016/j.cpr.2015.01.004 25638755

[15] Bes I Shoman Y Al-Gobari M Rousson V Guseva Canu I. Organizational interventions and occupational burnout: A meta-analysis with focus on exhaustion. Int Arch Occup Environ Health. (2023) 96:1211–23. doi: 10.1007/s00420-023-02009-z 37758838 PMC10560169

[16] Cohen C Pignata S Bezak E Tie M Childs J. Workplace interventions to improve well-being and reduce burnout for nurses, physicians and allied healthcare professionals: a systematic review. BMJ Open. (2023) 13:e071203. doi: 10.1136/bmjopen-2022-071203 37385740 PMC10314589

[17] Taylor C Maben J Jagosh J Carrieri D Briscoe S Klepacz N . Care under pressure 2: A realist synthesis of causes and interventions to mitigate psychological ill-health in nurses, midwives and paramedics. BMJ Qual Safety. (2024) 33:523–38. doi: 10.1136/bmjqs-2023-016468 38575309 PMC11287552

[18] Shoman Y Rousson V Bianchi R Guseva Canu I. Holistic assessment of factors associated with exhaustion, the main symptom of burnout: a meta-analysis of longitudinal studies. Int J Environ Res Public Health. (2022) 19:13037. doi: 10.3390/ijerph192013037 36293607 PMC9602979

[19] Rycroft-Malone J McCormack B Hutchinson AM DeCorby K Bucknall TK Kent B . Realist synthesis: illustrating the method for implementation research. Implementation Sci. (2012) 7:33. doi: 10.1186/1748-5908-7-33 22515663 PMC3514310

[20] Wong G Greenhalgh T Westhorp G Buckingham J Pawson R. RAMESES publication standards: realist syntheses. BMC Med. (2013) 11:21. doi: 10.1186/1741-7015-11-21 23360677 PMC3558331

[21] Moore GF Audrey S Barker M Bond L Bonell C Hardeman W . Process evaluation of complex interventions: Medical Research Council guidance. BMJ. (2015) 350:h1258. doi: 10.1136/bmj.h1258 25791983 PMC4366184

[22] Stone KW Kintziger KW Jagger MA Horney JA. Public health workforce burnout in the COVID-19 response. Int J Environ Res Public Health. (2021) 18:4369. doi: 10.3390/ijerph18084369 33924084 PMC8074254

[23] Scales SE Kintziger KW Stone KW Jagger MA Horney JA. Burnout among public health workers during the COVID-19 response: results from a follow-up survey. PLOS Mental Health. (2024) 1:e0000100. doi: 10.1371/journal.pmen.0000100 PMC12798593 41661732

[24] Kim M-N Yoo Y-S Cho O-H Hwang K-H. Emotional labor and burnout of public health nurses during the COVID-19 pandemic: mediating effects of perceived health status and perceived organizational support. Int J Environ Res Public Health. (2022) 19:549. doi: 10.3390/ijerph19010549 35010814 PMC8744956

[25] Baek S-U Yoon J-H Won J-U. Association between high emotional demand at work, burnout symptoms, and sleep disturbance among Korean workers: A cross-sectional mediation analysis. Sci Rep. (2023) 13:16688. doi: 10.1038/s41598-023-43451-w 37794088 PMC10550909

[26] Forte FDS Vieira NFC Farias SF . Quality of life and associated factors for community health workers in the context of the COVID-19 pandemic in northeastern Brazil. Sci Rep. (2024) 14:13312. doi: 10.1038/s41598-024-63828-9 38858430 PMC11164982

[27] Izdebski M Kozakiewicz A Białorudzki M Dec-Pietrowska J Mazur J. Occupational burnout in healthcare workers, stress and other symptoms of work overload during the COVID-19 pandemic in Poland. Int J Environm Res Public Health. (2023) 20:2428. doi: 10.3390/ijerph20032428 36767797 PMC9916221

[28] Maslach C Jackson SE. The measurement of experienced burnout. J Organ Behav. (1981) 2:99–113. doi: 10.1002/job.4030020205

[29] Morris JA Feldman DC. The dimensions, antecedents, and consequences of emotional labor. Acad Managem Rev. (1996) 21:986–1010. doi: 10.2307/259161

[30] Grandey AA. Emotion regulation in the workplace: A new way to conceptualize emotional labor. J Occup Health Psychol. (2000) 5:95–110. doi: 10.1037//1076-8998.5.1.95 10658889

[31] Brotheridge CM Grandey AA. Emotional labor and burnout: Comparing two perspectives of “people work.” *J Vocat Behav*. (2002) 60:17–39. doi: 10.1006/jvbe.2001.1815

[32] Zapf D. Emotion work and psychological well-being: A review of the literature and some conceptual considerations. Hum Resour Manag Rev. (2002) 12:237–68. doi: 10.1016/S1053-4822(02)00048-7

[33] Demerouti E Bakker AB Vardakou I Kantas A. The convergent validity of two burnout instruments: a multitrait–multimethod analysis. Eur J Psychol Assessm. (2003) 19:12–23. doi: 10.1027//1015-5759.19.1.12

[34] Shirom A. Reflections on the study of burnout. Work Stress. (2005) 19:263–70. doi: 10.1080/02678370500376649

[35] Halbesleben JRB. Sources of social support and burnout: a meta-analytic test of the conservation of resources model. J Appl Psychol. (2006) 91:1134–45. doi: 10.1037/0021-9010.91.5.1134 16953774

[36] Eisenberger R Huntington R Hutchison S Sowa D. Perceived organizational support. J Appl Psychol. (1986) 71:500–7. doi: 10.1037//0021-9010.71.3.500

[37] Rhoades L Eisenberger R. Perceived organizational support: a review of the literature. J Appl Psychol. (2002) 87:698–714. doi: 10.1037//0021-9010.87.4.698 12184574

[38] Kurtessis JN Eisenberger R Ford MT Buffardi LC Stewart KA Adis C. Perceived organizational support: a meta-analytic evaluation of organizational support theory. J Manage. (2017) 43:1854–84. doi: 10.1177/0149206315575554

[39] Edmondson A. Psychological safety and learning behavior in work teams. Admin Sci Quart. (1999) 44:350–83. doi: 10.2307/2666999

[40] Gerstner CR Day DV. Meta-analytic review of leader–member exchange theory: Correlates and construct issues. J Appl Psychol. (1997) 82:827–44. doi: 10.1037//0021-9010.82.6.827

[41] Skakon J Nielsen K Borg V Guzman J. Are leaders' well-being, behaviours and style associated with the affective well-being of their employees? A systematic review. Work Stress. (2010) 24:107–39. doi: 10.1080/02678373.2010.495262

[42] Thomas G Inceoglu I Chu C Plans D Gerbasi A. Leadership behavior and employee well-being: An integrated review and a future research agenda. Leadership Quart. (2018) 29:179–202. doi: 10.1016/j.leaqua.2017.12.006

[43] Xanthopoulou D Bakker AB Demerouti E Schaufeli WB. The role of personal resources in the Job Demands-Resources model. Int J Stress Manag. (2007) 14:121–41. doi: 10.1037/1072-5245.14.2.121

[44] Tims M Bakker AB Derks D. Development and validation of the Job Crafting Scale. J Vocat Behav. (2012) 80:173–86. doi: 10.1016/j.jvb.2011.05.009

[45] Anger WK Alley L Dimoff JK. Addressing health care workers' mental health: A systematic review of evidence-based interventions and current resources. Am J Public Health. (2024) 114:213–26. doi: 10.2105/AJPH.2023.307556 38354343 PMC10916736

[46] Cooklin A Joss N Husser E Oldenburg B. Integrated approaches to occupational health and safety: a systematic review. Am J Health Promot. (2017) 31:401–12. doi: 10.4278/ajhp.141027-LIT-542 26730561

[47] Feltner C Peterson K Palmieri Weber R Cluff L Coker-Schwimmer E Viswanathan M . The effectiveness of Total Worker Health^®^ interventions: a systematic review. Ann Intern Med. (2016) 165:262–9. doi: 10.7326/M16-0626 27240022

[48] Gray P Senabe S Naicker N Kgalamono S Yassi A Spiegel JM. Workplace-based organizational interventions promoting mental health and happiness among healthcare workers: a review. Int J Environ Res Public Health. (2019) 16:4396. doi: 10.3390/ijerph16224396 31717906 PMC6888154

[49] Roczniewska M Agerström J Kecklund G Holmström E. Job-focused intervention studies: a systematic review and meta-analysis of randomized controlled trials of job crafting and related workplace interventions. Syst Rev. (2023) 12:45. doi: 10.1186/s13643-023-02170-z36918977

[50] Sakuraya A Shimazu A Imamura K Kawakami N. Effects of a job crafting intervention program on work engagement among Japanese employees: a randomized controlled trial. Front Psychol. (2020). doi: 10.3389/fpsyg.2020.00235 32153460 PMC7047874

[51] Nebbs A Martin A Neil A Dawkins S Roydhouse J. An integrated approach to workplace mental health: a scoping review of instruments that can assist organizations with implementation. Int J Environ Res Public Health. (2023) 20:1192. doi: 10.3390/ijerph20021192 36673948 PMC9859114

[52] Dennerlein JT Burke L Sabbath EL . An integrative Total Worker Health framework for keeping workers safe and healthy during the COVID-19 pandemic. Hum Factors. (2020) 62:689–96. doi: 10.1177/0018720820932699 32515231 PMC7346710

[53] Nielsen K Christensen M. Positive participatory organizational interventions: a multilevel approach for creating healthy workplaces. Front Psychol. (2021) 12:696245. doi: 10.3389/fpsyg.2021.696245 34262513 PMC8273334

[54] Arapovic-Johansson B Jensen I Wåhlin C Björklund C Kwak L. Process evaluation of a participative organizational intervention as a stress-preventive intervention for employees in Swedish primary health care. Int J Environ Res Public Health. (2020) 17:7285. doi: 10.3390/ijerph17197285 33036154 PMC7579215

[55] Bakker AB Demerouti E. The Job Demands-Resources model: state of the art. J Manag Psychol. (2007) 22:309–28. doi: 10.1108/02683940710733115

[56] Yang C Chen Y Zhao X. Emotional labor: scale development and validation in the Chinese context. Front Psychol. (2019) 10:2095. doi: 10.3389/fpsyg.2019.02095 31620048 PMC6759872

[57] Maslach C Jackson SE Leiter MP. Maslach Burnout Inventory Manual (3rd ed.). Washington, DC: Consulting Psychologists Press (2001).

